# No evidence of disease activity (NEDA) analysis by epochs in patients with relapsing multiple sclerosis treated with ocrelizumab vs interferon beta-1a

**DOI:** 10.1177/2055217318760642

**Published:** 2018-03-12

**Authors:** Eva Havrdová, Douglas L Arnold, Amit Bar-Or, Giancarlo Comi, Hans-Peter Hartung, Ludwig Kappos, Fred Lublin, Krzysztof Selmaj, Anthony Traboulsee, Shibeshih Belachew, Iain Bennett, Regine Buffels, Hideki Garren, Jian Han, Laura Julian, Julie Napieralski, Stephen L Hauser, Gavin Giovannoni

**Affiliations:** Department of Neurology and Center for Clinical Neuroscience, Charles University, Czech Republic; Department of Neurology and Neurosurgery, McGill University, Canada; NeuroRx Research, Canada; Department of Neurology and Center for Neuroinflammation and Experimental Therapeutics, University of Pennsylvania, USA; Neurology Department and INSPE-Institute of Experimental Neurology, Vita-Salute San Raffaele University, Italy; Medical Faculty, Heinrich Heine University Düsseldorf, Germany; Neurologic Clinic and Policlinic, Departments of Medicine, Clinical Research, Biomedicine and Biomedical Engineering, University Hospital Basel, Switzerland; Department of Neurology, Icahn School of Medicine at Mount Sinai, USA; Medical University of Lodz, Poland; Division of Neurology, Department of Medicine, University of British Columbia, Canada; F. Hoffmann-La Roche Ltd, Switzerland; F. Hoffmann-La Roche Ltd, Switzerland; F. Hoffmann-La Roche Ltd, Switzerland; Genentech Inc, USA; Genentech Inc, USA; Genentech Inc, USA; F. Hoffmann-La Roche Ltd, Switzerland; Department of Neurology University of California, San Francisco, USA; Blizard Institute, Barts and The London School of Medicine and Dentistry, Queen Mary University London, UK

**Keywords:** NEDA, disease activity, relapse, disability progression, MRI activity

## Abstract

**Background:**

No evidence of disease activity (NEDA; defined as no 12-week confirmed disability progression, no protocol-defined relapses, no new/enlarging T2 lesions and no T1 gadolinium-enhancing lesions) using a fixed-study entry baseline is commonly used as a treatment outcome in multiple sclerosis (MS).

**Objective:**

The objective of this paper is to assess the effect of ocrelizumab on NEDA using re-baselining analysis, and the predictive value of NEDA status.

**Methods:**

NEDA was assessed in a modified intent-to-treat population (*n* = 1520) from the pooled OPERA I and OPERA II studies over various epochs in patients with relapsing MS receiving ocrelizumab (600 mg) or interferon beta-1a (IFN β‐1a; 44 μg).

**Results:**

NEDA was increased with ocrelizumab vs IFN β-1a over 96 weeks by 75% (*p* < 0.001), from Week 0‒24 by 33% (*p* < 0.001) and from Week 24‒96 by 72% (*p* < 0.001). Among patients with disease activity during Weeks 0‒24, 66.4% vs 24.3% achieved NEDA during Weeks 24‒96 in the ocrelizumab and IFN β-1a groups (relative increase: 177%; *p* < 0.001).

**Conclusion:**

Superior efficacy with ocrelizumab compared with IFN β-1a was consistently seen in maintaining NEDA status in all epochs evaluated. By contrast with IFN β-1a, the majority of patients with disease activity early in the study subsequently attained NEDA status with ocrelizumab.

## Introduction

No evidence of disease activity (NEDA) is a composite measure of the absence of confirmed disability worsening (as measured by the Expanded Disability Status Scale (EDSS)) and of clinical and magnetic resonance imaging (MRI) measures of disease activity in relapsing multiple sclerosis (RMS). NEDA was originally described in a post-hoc analysis of the placebo-controlled, two-year Phase III pivotal trial for natalizumab.^1^ NEDA has since been widely used in analyses of other disease-modifying therapies (DMTs),^2‒9^ and has been adopted as an outcome measure in RMS clinical trials.^10–13^ NEDA may provide a more sensitive and comprehensive measure of capturing overall treatment benefit and has been proposed as a primary endpoint in future pivotal Phase III clinical studies.^14,15^ NEDA is increasingly recognized as an important treatment goal for patients with RMS^16^ and has been shown to be informative in the prediction of long-term disability progression both independent of DMT type^17^ (cohort study) and in the core and extension periods of clinical trial patient populations.^18^

As a binary outcome, NEDA status and its failure are often driven by MRI components of the measure and hence are particularly influenced by the frequency of MRI assessments.^8,14^ The analysis of NEDA is also affected by the pharmacodynamics of the particular therapy assessed. Re-baselining, wherein a post-study baseline time point is utilized as the new baseline reference for subsequent assessment of disability worsening and disease activity, therefore may reflect a truer representation of a DMT’s steady state of efficacy^16^ unconfounded by any initial disease activity carried over from baseline and recent pre-baseline disease state.

B cells are a significant contributor to the pathogenesis of MS.^19,20^ CD20 is a cell-surface antigen expressed on pre-B cells, mature B cells and memory B cells, but not lymphoid stem cells and plasma cells.^21‒23^ Ocrelizumab is a recombinant, humanized monoclonal antibody that selectively depletes CD20-expressing B cells^24,25^ while preserving the capacity for B-cell reconstitution and maintaining pre-existing humoral immunity.^26,27^ In the two identical Phase III trials, OPERA I and OPERA II, ocrelizumab significantly reduced all individual components of NEDA compared with high-dose, high-frequency interferon beta-1a (IFN β‐1a) at Week 96 in patients with RMS.^28^ The objective of the current analyses was to assess the effect of ocrelizumab on the proportion of patients with NEDA and determine the predictive value of NEDA over time in multiple epochs across the pooled OPERA I and OPERA II studies.

## Patients and methods

### Trial design and patients

NEDA was determined in the pooled population of the two identical Phase III, multicenter, randomized, double-blind, double-dummy, parallel-group OPERA I and OPERA II trials (OPERA I/NCT01247324 and OPERA II/NCT01412333). The study protocol was approved by each center’s independent ethics committee. The study design was written in accordance with the Declaration of Helsinki and Good Clinical Practice guidelines, and all enrolled patients provided written informed consent. The OPERA I and OPERA II trials were deemed poolable based on their identical protocols and according to formal poolability testing results.^28^ Baseline demographics and disease characteristics were not different within each study arm or between studies. Study details have been reported previously.^28^ Key eligibility criteria included an age of 18 to 55 years, diagnosis of RMS (2010 revised McDonald criteria)^29^ and an EDSS score of 0 to 5.5 at screening. Patients were randomized (1:1) to receive either ocrelizumab 600 mg by intravenous infusion every 24 weeks or subcutaneous IFN β-1a three times per week at a dose of 44 μg throughout the 96-week treatment period (including a per-label incremental titration scheme in the first four weeks). Patients were stratified by region (United States (US) vs rest of world (ROW)) and baseline EDSS score (<4.0 and ≥4.0) in the randomization for OPERA I and OPERA II.

### Clinical and MRI endpoints, including NEDA

EDSS scores were determined at screening, baseline and every 12 weeks: Confirmed disability progression (CDP) was defined as a ≥1-point increase in EDSS score from a baseline EDSS score of 0–5.5, or a 0.5-point increase in EDSS score from a baseline EDSS score greater than 5.5, sustained for at least 12 weeks (12-week CDP). Protocol-defined relapses were new or worsening neurological symptoms attributable to MS: Symptoms must (i) persist for greater than 24 hours, should not be attributable to confounding clinical factors, and be immediately preceded by a stable or improving neurological state for at least 30 days; and (ii) be accompanied by objective neurological worsening consistent with an increase of at least half a step on the EDSS scale, or two points on at least one of the appropriate Functional Systems Scores (FSSs), or one point on two or more of the appropriate FSSs. Brain MRI was performed at baseline and Weeks 24, 48 and 96; new or enlarging T2 lesions and/or T1 gadolinium-enhancing lesions on any scan during the epoch investigated were considered evidence of MRI disease activity. NEDA status was defined as the combined absence of: protocol-defined relapses; 12-week CDP; new or enlarging T2 lesions; and T1 gadolinium-enhancing lesions.

### Statistical analyses

NEDA outcome was primarily assessed in a modified intent-to-treat (mITT) population over the controlled treatment phase (baseline to 96 weeks),^28^ which included all patients in the intent-to-treat population, but patients who discontinued treatment early for reasons other than lack of efficacy or death and had NEDA before early discontinuation were excluded. Further analyses evaluated the proportion of patients with NEDA for several epochs, including: Weeks 0–48; Weeks 48–96 (where all components of NEDA including 12-week CDP were re-baselined to Week 48); Weeks 0–24; Weeks 24–48 (where all components of NEDA including 12-week CDP were re-baselined to Week 24); and Weeks 24–96 (where all components of NEDA including 12-week CDP were re-baselined to Week 24). NEDA and its components were compared in patients treated with ocrelizumab with those receiving IFN β-1a in a post-hoc analysis using the Cochran–Mantel–Haenszel test stratified by study, geographic region (US vs ROW) and baseline EDSS score (<4.0 vs ≥4.0). Patients who discontinued treatment early with at least one event (i.e. protocol-defined relapse, 12-week CDP, new or enlarging T2 lesion or T1 gadolinium-enhancing lesion) before early discontinuation were considered as having evidence of disease activity (EDA). Even if a patient did not report an event before early discontinuation, the patient was considered as having EDA if the reason for early discontinuation was lack of efficacy or death.

### Probability of attaining or maintaining NEDA relative to earlier EDA or NEDA status, and the predictive value of NEDA status

Subgroups of patients by NEDA status in Weeks 0–24 were assessed for subsequent NEDA status in Weeks 24–96 and in Weeks 24–48. Similarly, subgroups of patients by NEDA status in Weeks 24–48 were assessed for subsequent NEDA status in Weeks 48–96. Using the pooled treatment arms, NEDA status in Weeks 0–48 was used to predict time to (i) first protocol-defined relapse, and (ii) first 12-week and 24-week CDP in Weeks 48–96, and was evaluated based on the Cox proportional hazard regression model which included the NEDA during Weeks 0–48 as a factor, stratified by study, geographical region (US vs ROW) and baseline EDSS score (<4.0 vs ≥4.0).

## Results

### Patient demographics and disease characteristics

In the OPERA I and OPERA II trials, the pooled intention-to-treat population comprised 1656 patients (IFN β-1a, *n* = 829; ocrelizumab, *n* = 827). The mITT reference population used for the NEDA analysis comprised 759 and 761 patients randomized to high-dose, high-frequency IFN β-1a and ocrelizumab, respectively. Detailed baseline demographics and disease characteristics of (i) the mITT population and (ii) patients with EDA and NEDA over 96 weeks are presented by treatment arm in Table 1. All major baseline covariates were balanced between treatment groups in the mITT population. Compared with patients who maintained NEDA over 96 weeks, patients with EDA across treatment groups had slightly more relapses recorded in the 12 months prior to baseline, a greater number of T1 gadolinium-enhanced lesions and a higher T2 lesion burden at baseline. The age and proportion of female patients were slightly lower in the EDA group. Baseline EDSS score, disease duration and normalized brain volume were similar between patients who maintained NEDA or experienced EDA events.

**Table 1.** Patient baseline demographics and disease characteristics of the total pooled mITT patient population, and of patients maintaining NEDA vs having EDA over 96 weeks.

Baseline demographics and disease characteristics	Pooled mITT population		Patients with NEDA		Patients with EDA	
	IFN β-1a*N* = 759	Ocrelizumab*N* = 761	IFN β-1a*N* = 206	Ocrelizumab*N* = 363	IFN β-1a*N* = 553	Ocrelizumab*N* = 398
Age, mean (SD), years	37.1 (9.2)	37.2 (9.1)	39.4 (9.3)	38.4 (9.0)	36.2 (9.0)	36.1 (9.1)
Female, % (*n*)	66.8 (507)	64.8 (493)	72.8 (150)	67.5 (245)	64.6 (357)	62.3 (248)
Time since MS symptom onset, mean (SD), years	6.4 (6.1)	6.6 (6.1)	6.1 (6.1)	6.8 (6.3)	6.5 (6.1)	6.4 (5.9)
Time since MS diagnosis, mean (SD), years	3.9 (4.8)	3.9 (4.9)	3.5 (4.8)	4.1 (5.1)	4.0 (4.9)	3.8 (4.6)
Number of relapses in previous 12 months, mean (SD)	1.3 (0.7)^a^	1.3 (0.7)	1.3 (0.6)	1.3 (0.6)	1.4 (0.7)^b^	1.4 (0.7)
EDSS score, mean (SD)	2.8 (1.3)	2.8 (1.3)	2.8 (1.3)	2.7 (1.2)	2.8 (1.3)	2.9 (1.3)
MRI						
Number of T1 Gd^+^ lesions, mean (SD)	2.0 (5.2)^c^	1.8 (4.7)^d^	0.4 (1.4)^e^	0.7 (1.8)^f^	2.6 (5.9)^g^	2.8 (6.1)^h^
Brain T2 hyperintense lesion volume, median (range), cm^3^	6.2 (0–76.1)^i^	5.4 (0–96.0)^j^	3.8 (0–60.2)^k^	3.8 (0–96.0)^l^	7.2 (0.1–76.1)^m^	7.5 (0–83.2)^n^
Normalized brain volume, mean (SD), cm^3^	1499.8 (88.3)^o^	1501.7 (88.1)^p^	1502.3 (84.1)^q^	1502.3 (90.3)^r^	1498.9 (89.8)^s^	1501.3 (86.2)^t^

EDA: evidence of disease activity; EDSS: Expanded Disability Status Scale; Gd^+^: gadolinium-enhancing; IFN β-1a: interferon beta-1a; mITT: modified intent-to-treat; MRI: magnetic resonance imaging; MS: multiple sclerosis; NEDA: no evidence of disease activity; SD: standard deviation.

^a^*n* = 757; ^b^*n* = 551; ^c^*n* = 752; ^d^*n* = 753; ^e^*n* = 205; ^f^*n* = 357; ^g^*n* = 547; ^h^*n* = 396; ^i^*n* = 754; ^j^*n* = 756; ^k^*n* = 205; ^l^*n* = 359; ^m^n=549; ^n^*n* = 397; ^o^*n* = 749; ^p^*n* = 754; ^q^*n* = 204; ^r^*n* = 357; ^s^*n* = 545; ^t^*n* = 397.

### Overall NEDA (Week 0–96) and re-baselined NEDA (Weeks 24–96) status in the pooled OPERA I and OPERA II studies

**Figure 1.**
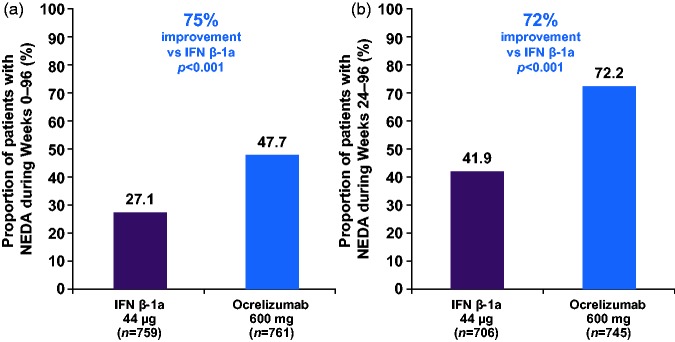
Proportion of patients with NEDA during (a) Weeks 0–96, and (b) during Weeks 24–96, re-baselined to Week 24. Compared using the Cochran–Mantel–Haenszel test stratified by study, geographic region (United States vs rest of world) and baseline EDSS score (<4.0 vs ≥4.0). Weeks 24–96: MRI at Week 48, Week 96 and unscheduled post-Week 24 scans prior to Week 96 are used in the definition of NEDA; this implies that analysis of the Week 24–96 epoch is based on two MRI scans. Weeks 24–96: Data were re-baselined to Week 24, i.e. all components of NEDA including 12-week CDP are defined relative to Week 24. CDP: confirmed disability progression; EDSS: Expanded Disability Status Scale; IFN β-1a: interferon beta-1a; MRI: magnetic resonance imaging; NEDA: no evidence of disease activity.

In the pooled analyses of OPERA I and OPERA II, the relative proportion of patients with NEDA was increased by 75% (47.7% vs 27.1%; *p* < 0.001) with ocrelizumab compared with IFN β-1a over 96 weeks (Figure 1(a)).^28^ Following re-baselining at Week 24, the relative proportion of patients with NEDA was 72% higher (72.2% vs 41.9%; *p* < 0.001) with ocrelizumab compared with IFN β-1a during Weeks 24–96 (Figure 1(b)).

During the 96-week epoch, a significant difference in the proportion of patients without disease activity was seen for each individual component of NEDA, including 12-week CDP, with ocrelizumab compared with IFN β-1a (*p* < 0.001; Table 2). This was reflected in the proportion of patients with no disability worsening and clinical disease activity (no 12-week CDP and no relapses: ocrelizumab 73.8% vs IFN β-1a 59.4%; *p* < 0.001) and no brain MRI measures of disease activity (no new or enlarging T2 lesions and no T1 gadolinium-enhancing lesions: ocrelizumab 62.2% vs IFN β-1a 37.6%; *p* < 0.001; Table 2). Following re-baselining at Week 24, similar results were seen during Weeks 24–96 for the individual components of NEDA and the pairwise combination of clinical and MRI measures (Table 2).

**Table 2.** Proportion of patients with NEDA (and its individual components) in all epochs of the pooled OPERA I and OPERA II studies (mITT population).

	Study epoch (weeks)											
	0–96		0–24		0–48		24–48^a^		24–96^a^		48–96^b^	
	IFN β-1a	OCR	IFN β-1a	OCR	IFN β-1a	OCR	IFN β-1a	OCR	IFN β-1a	OCR	IFN β-1a	OCR
Proportion of patients with NEDA, %	27.1	47.7	45.7	60.8	34.9	54.6	59.0	85.8	41.9	72.2	57.3	81.8
(*n*/*N*)^c^	(206/759)	(363/761)	(356/779)	(478/786)	(268/769)	(424/777)	(429/727)	(662/772)	(296/706)	(538/745)	(388/677)	(602/736)
Relative risk (CI)	1.75 (1.53–2.01)		1.33 (1.21–1.46)		1.56 (1.39–1.76)		1.45 (1.36–1.55)		1.72 (1.56–1.90)		1.43 (1.33–1.54)	
*p* value	*p* < 0.001		*p* < 0.001		*p* < 0.001		*p* < 0.001		*p* < 0.001		*p* < 0.001	
Proportion of patients with no CDP and no relapses, %	59.4	73.8	83.2	88.9	70.8	81.8	81.1	89.1	66.2	76.5	76.3	83.3
(*n/N*)	(431/726)	(556/753)	(624/750)	(676/760)	(522/737)	(619/757)	(579/714)	(673/755)	(461/696)	(569/744)	(514/674)	(613/736)
Relative risk (CI)	1.25 (1.16–1.34)		1.07 (1.03–1.11)		1.16 (1.09–1.22)		1.10 (1.05–1.15)		1.16 (1.08–1.24)		1.10 (1.04–1.15)	
*p* value	*p* < 0.001		*p* = 0.001		*p* < 0.001		*p* < 0.001		*p* < 0.001		*p* < 0.001	
Proportion of patients with no relapses, %	67.3	80.0	87.3	92.5	77.4	87.6	87.2	94.4	75.1	85.4	83.9	89.4
(*n*/*N*)	(511/759)	(609/761)	(680/779)	(727/786)	(595/769)	(681/777)	(634/727)	(729/772)	(530/706)	(636/745)	(568/677)	(659/737)
Relative risk (CI)	1.19 (1.12–1.26)		1.06 (1.02–1.10)		1.13 (1.08–1.19)		1.08 (1.05–1.12)		1.14 (1.08–1.20)		1.07 (1.02–1.11)	
*p* value	*p* < 0.001		*p* < 0.001		*p* < 0.001		*p* < 0.001		*p* < 0.001		*p* < 0.001	
Proportion of patients with no CDP, %	82.4	88.7	95.3	96.1	91.2	92.9	93.0	94.6	86.0	89.1	90.5	92.8
(*n*/*N*)	(625/759)	(675/761)	(742/779)	(755/786)	(701/769)	(722/777)	(676/727)	(730/772)	(607/706)	(664/745)	(613/677)	(683/736)
Relative risk (CI)	1.08 (1.03–1.12)		1.01 (0.99–1.03)		1.02 (0.99–1.05)		1.02 (0.99–1.04)		1.04 (1.00–1.08)		1.03 (1.00–1.06)	
*p* value	*p* < 0.001		*p* = 0.42		*p* = 0.18		*p* = 0.19		*p* = 0.057		*p* = 0.082	
Proportion of patients with no brain MRI activity, %	37.6	62.2	51.6	66.1	43.8	63.7	67.8	95.2	53.6	92.7	69.9	97.0
(*n*/*N*)	(279/742)	(469/754)	(389/754)	(502/760)	(328/749)	(484/760)	(485/715)	(720/756)	(376/701)	(688/742)	(472/675)	(713/735)
Relative risk (CI)	1.65 (1.48–1.84)		1.28 (1.17–1.39)		1.45 (1.32–1.60)		1.40 (1.33–1.48)		1.73 (1.61–1.86)		1.39 (1.32–1.46)	
*p* value	*p* < 0.001		*p* < 0.001		*p* < 0.001		*p* < 0.001		*p* < 0.001		*p* < 0.001	
Proportion of patients with no new or enlarging T2 lesions, %	39.1	62.8	54.0	67.4	46.7	64.9	69.7	95.9	56.1	94.5	71.5	98.2
(*n*/*N*)	(297/759)	(478/761)	(421/779)	(530/786)	(359/769)	(504/777)	(506/727)	(740/772)	(396/706)	(704/745)	(484/677)	(723/736)
Relative risk (CI)	1.60 (1.44–1.78)		1.25 (1.15–1.35)		1.39 (1.27–1.52)		1.38 (1.31–1.45)		1.68 (1.57–1.80)		1.37 (1.31–1.44)	
*p* value	*p* < 0.001		*p* < 0.001		*p* < 0.001		*p* < 0.001		*p* < 0.001		*p* < 0.001	
Proportion of patients with no T1 Gd^+^ lesions, %	69.7	95.0	84.7	97.7	78.0	96.9	86.1	98.8	78.6	98.5	86.7	99.5
(*n*/*N*)	(529/759)	(723/761)	(660/779)	(768/786)	(600/769)	(753/777)	(626/727)	(763/772)	(555/706)	(734/745)	(587/677)	(733/737)
Relative risk (CI)	1.36 (1.30–1.43)		1.15 (1.12–1.19)		1.24 (1.19–1.29)		1.15 (1.11–1.18)		1.25 (1.21–1.30)		1.15 (1.11–1.18)	
*p* value	*p* < 0.001		*p* < 0.001		*p* < 0.001		*p* < 0.001		*p* < 0.001		*p* < 0.001	

CDP: confirmed disability progression; CI: confidence interval; Gd^+^: gadolinium-enhancing; IFN β-1a: interferon beta-1a; mITT: modified intent-to-treat; MRI: magnetic resonance imaging; NEDA: no evidence of disease activity; OCR: ocrelizumab.

^a^Weeks 24–48 and 24–96: Data were re-baselined to Week 24, i.e. all components of NEDA including 12-week CDP are defined relative to Week 24. ^b^Weeks 48–96: Data were re-baselined to Week 48, i.e. all components of NEDA including 12-week CDP are defined relative to Week 48. ^c^*n*/*N*: *n* is the number of patients maintaining “no event” status for respective endpoints in the table; proportions are based on *N*.

### NEDA status analysis in all epochs of OPERA studies

Irrespective of the various epochs studied, a favorable outcome was seen with ocrelizumab compared with IFN β-1a; the relative proportion of patients with NEDA was 56% higher (54.6% vs 34.9%) with ocrelizumab during Weeks 0–48, 43% higher (81.8% vs 57.3%) during Weeks 48–96, 33% higher (60.8% vs 45.7%) during Weeks 0–24 and 45% higher (85.8% vs 59.0%) during Weeks 24–48 (all comparisons *p* < 0.001; Table 2). Sensitivity analyses of the relative proportion of patients with NEDA during Weeks 0–48 excluding T1 gadolinium-enhancing lesions at Week 24 revealed similar results to the original analysis (Supplementary Materials Table S1).

### Probability of attaining or maintaining NEDA relative to earlier EDA or NEDA status

**Figure 2.**
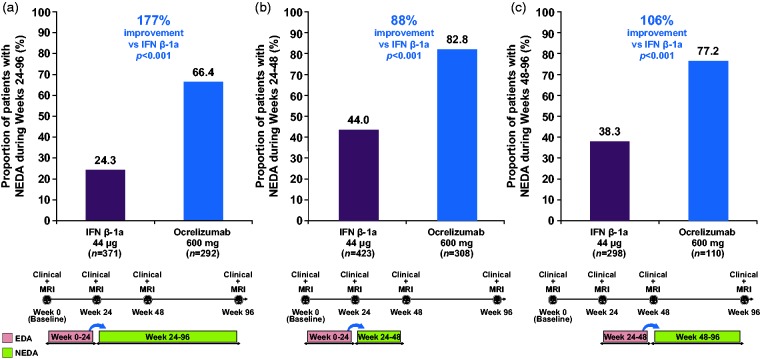
Proportion of patients with NEDA during (a) Weeks 24–96, (b) Weeks 24–48 among patients with EDA in Weeks 0–24 and (c) Weeks 48–96 among patients with EDA in Weeks 24–48. All components of NEDA including 12-week CDP are defined relative to Week 24 (a) and (b), and relative to Week 48 (c). Comparison using the Cochran–Mantel–Haenszel test stratified by study, geographic region (United States vs rest of world) and baseline EDSS score (<4.0 vs ≥4.0). CDP: confirmed disability progression; EDA: evidence of disease activity; EDSS: Expanded Disability Status Scale; IFN β-1a: interferon beta-1a; MRI: magnetic resonance imaging; NEDA: no evidence of disease activity; EDA: evidence of disease activity.

In the ocrelizumab and IFN β-1a groups respectively, 66.4% vs 24.3% of patients with EDA during Weeks 0–24 subsequently attained NEDA status during Weeks 24–96 (relative increase with ocrelizumab: 177%; *p* < 0.001, Figure 2(a)), and 82.8% vs 44.0% attained NEDA status during Weeks 24–48 (relative increase: 88%; *p* < 0.001, Figure 2(b)). Among patients with NEDA during Weeks 0–24, the proportion of patients who maintained NEDA during Weeks 24–96 was 75.9% vs 61.5% in the ocrelizumab and IFN β-1a groups, respectively (relative increase with ocrelizumab: 23%; *p* < 0.001), and during Weeks 24–48 the proportion was 87.6% vs 75.7% (relative increase with ocrelizumab: 16%; *p* < 0.001). Of patients with EDA during Weeks 24–48, in the ocrelizumab and IFN β-1a groups respectively, 77.2% vs 38.3% subsequently attained NEDA status during Weeks 48–96 (relative increase with ocrelizumab: 106%; *p* < 0.001, Figure 2(c)); among those patients with NEDA during Weeks 24–48, the proportion of patients who maintained NEDA during Weeks 48–96 was 82.5% vs 69.9% (relative increase with ocrelizumab: 18%; *p* < 0.001).

### Predictive value of first-year NEDA status for subsequent risk of relapse and disability progression

When the treatment arms were pooled, patients with NEDA during Weeks 0–48 had a subsequent (Weeks 48–96) 53% risk reduction (hazard ratio (HR): 0.47; 95% confidence interval (CI): 0.35–0.65; *p* < 0.001) in time to first relapse, a 36% risk reduction (HR: 0.64; 95% CI: 0.44–0.94; *p* = 0.023) in time to first 12-week CDP, and a 39% risk reduction (HR: 0.61; 95% CI: 0.39–0.95; *p* = 0.028) in time to first 24-week CDP, compared with those patients with EDA during Weeks 0–48.

## Discussion

A higher proportion of patients had NEDA with ocrelizumab at the first MRI at Week 24 and in all epochs of the OPERA studies compared with high-dose, high-frequency IFN β-1a. Over two years, 48% of patients treated with ocrelizumab had NEDA, and as many as 72% had NEDA in Weeks 24–96 and 82% in Weeks 48–96, which is greater than that observed with other high-efficacy therapies in similar epochs.^1,30,31^ Absolute proportions of patients maintaining NEDA status should be considered in the context of the existence of a certain level of inherent background noise in the binary assessment of NEDA status because of potential false-positive detection of clinical EDA events, particularly for relapses, which may yield a treatment ceiling effect. Pairwise combinations of disease parameters in the clinical and MRI components of NEDA showed the consistent overall benefit of ocrelizumab compared with IFN β-1a, which was further reflected in all the individual components of NEDA, including confirmed disability progression. Compared with patients who maintained NEDA over 96 weeks, the patients with EDA across treatment groups had higher brain MRI activity at baseline as measured by T1 gadolinium-enhancing lesions and higher T2 lesion burden, while no sizable difference was seen in pre-baseline relapse rate, and baseline EDSS score, disease duration and brain atrophy.

The early impact of ocrelizumab on MS disease activity was shown in a Phase II, randomized, placebo-controlled, double-blind trial in patients with relapsing–remitting MS, in which ocrelizumab demonstrated a robust effect on MRI activity as early as Week 8 after initiating treatment.^32^ However, in order to represent the full efficacy of a DMT unconfounded by disease activity carried over during the first four to eight weeks from treatment initiation, particularly MRI related, a re-baselining approach at first available MRI has been suggested.^3,14,33,34^ Re-baselining MS disease parameters to Week 24 showed a 72% relative increase in the proportion of ocrelizumab-treated patients with NEDA from Weeks 24 to 96 compared with IFN β-1a-treated patients. Moving into the clinical practice setting, the optimal timing of re-baselining should reflect the anticipated timing for reaching complete DMT efficacy, to give a more reliable indication of subsequent drug failure. Conclusions from cross-trial comparisons are limited because of differences including comparators, patient populations, MRI techniques, frequency of assessments, analysis methods and definitions of NEDA. Nevertheless, irrespective of the epoch chosen (including baseline–Week 24, baseline–Week 48 and Weeks 24–96), greater absolute proportions of patients with NEDA were observed in the present analysis compared with those reported with other high-efficacy therapies for RMS.^1,8,10,33^

As noted above and reported in other studies,^14,35^ the lower frequency of MRI scans during Weeks 48–96 compared with the other epochs described may have influenced the proportions of patients maintaining NEDA. However, despite the longer duration and the additional (Week 48) scan within the NEDA analysis of the Week 24–96 epoch, the proportion of patients maintaining NEDA in the ocrelizumab group was higher (72%) from Weeks 24–96 than from Weeks 0–24 (61%), while the reverse was seen in the IFN β-1a group.

The majority of patients with EDA in Weeks 0–24 or Weeks 24–48 subsequently attained NEDA in Weeks 24–96 and Weeks 48–96 with ocrelizumab treatment, whereas such patients mainly continued to experience disease activity with IFN β‐1a. Delayed conversion to NEDA status in patients with EDA in the first few months of treatment has been similarly reported for other high-efficacy DMTs,^1,31,33^ findings that argue against the immediate discontinuation of such therapies based on early signs of EDA, although longer-term confirmation of maintenance of NEDA beyond Week 96 warrants further investigation in the open-label extension study.

When pooling treatment arms, NEDA status in the first year (Weeks 0–48) predicted a lower risk of relapse (Kaplan–Meier analysis of time to first relapse) and a lower risk of disability progression (as measured by 12- and 24-week CDP) in the second year (Weeks 48–96). The lower risk of subsequent relapse and disability worsening in patients with NEDA during the first year, combined with the high proportion of patients receiving ocrelizumab treatment maintaining NEDA in Year 2 in those patients with NEDA in Year 1, suggest NEDA status over the short term may predict longer-term benefits. Contradictory data have been reported on the prognostic value of NEDA status over two years in predicting future disability progression up to 10 years, at least in patient cohorts from real-world settings where the majority of patients were treated with self-injectable DMTs (interferons or glatiramer acetate).^17,36,37^ Few patients maintained NEDA over the long term in studies in which self-injectable DMTs were used (7.9% over seven years;^17^ 0% over 10 years^9^). Conversely, NEDA was enhanced in patients over the long term in studies in which more effective DMTs were used (34% over seven years with natalizumab;^38^ 40% over five years with alemtuzumab^39^). These data support the notion that, balanced with any risk associated with a particular DMT, NEDA may be a realizable long-term treatment goal for patients with RMS in the era of higher-efficacy therapies. Furthermore, re-baselining of NEDA status especially during the first year of DMT initiation might have value in clinical practice to assess early treatment response, and inform longer-term therapeutic decisions to optimize the control of MS disease activity.

Further evolution of the NEDA concept may include the future integration of brain volume loss, and research is ongoing to determine the optimal threshold(s) of annualized rates that may discriminate pathological atrophy at the individual patient level, while accounting for the effect of aging.^9,40^ Similarly, the incorporation of cognitive, ambulation and upper extremity function measures may enable a more comprehensive ascertainment of the absence of disability progression when assessing NEDA.

Overall, ocrelizumab consistently resulted in a profound reduction of clinical and subclinical disease activity compared with IFN β-1a in patients with RMS, as measured by NEDA across various epochs. Understanding the associations between NEDA and patient-reported outcomes is warranted to better inform the day-to-day relevance of maintaining NEDA status. Data from open-label extension studies will help determine whether NEDA maintained in the two-year OPERA studies will translate into sustained NEDA and enhanced protection against accrual of disability over the long term.

## Supplemental Material

Supplemental material for No evidence of disease activity (NEDA) analysis by epochs in patients with relapsing multiple sclerosis treated with ocrelizumab vs interferon beta-1a Click here for additional data file. Supplemental material for No evidence of disease activity (NEDA) analysis by epochs in patients with relapsing multiple sclerosis treated with ocrelizumab vs interferon beta-1a by Eva Havrdová, Douglas L Arnold, Amit Bar-Or, Giancarlo Comi, Hans-Peter Hartung, Ludwig Kappos, Fred Lublin, Krzysztof Selmaj, Anthony Traboulsee, Shibeshih Belachew, Iain Bennett, Regine Buffels, Hideki Garren, Jian Han, Laura Julian, Julie Napieralski, Stephen L Hauser and Gavin Giovannoni in Multiple Sclerosis Journal – Experimental, Translational and Clinical

## Trial registration

ClinicalTrials.gov identifiers: OPERA I/NCT01247324 and OPERA II/NCT01412333. https://clinicaltrials.gov/ct2/show/NCT01247324https://clinicaltrials.gov/ct2/show/NCT01412333
