# Supplementary material for: No evidence of disease activity (NEDA) analysis by epochs in patients with relapsing multiple sclerosis treated with ocrelizumab vs interferon beta-1a
Source: Mult Scler J Exp Transl Clin. 2018 Mar 12;4(1):2055217318760642. doi: 10.1177/2055217318760642 (PMC5858626; doi:10.1177/2055217318760642)
Supplement: Supplemental material for No evidence of disease activity (NEDA) analysis by epochs in patients with relapsing multiple sclerosis treated with ocrelizumab vs interferon beta-1a [file Supplemental_material.pdf]

Supplementary Materials

Table S1. Sensitivity analyses of the relative proportion of patients with NEDA during Weeks 0–48 excluding the assessment of MRI T1 gadolinium-enhancing lesions at Week 24.

	Week 0–48 primary analysis		Week 0–48 sensitivity analysis excluding Week 24 T1 Gd^{+} assessment	
	**IFN β-1a**	**OCR**	**IFN β-1a**	**OCR**
**Proportion of patients with NEDA, %**	34.9	54.6	34.9	54.8
(n/N)	(268/769)	(424/777)	(268/769)	(426/777)
Relative risk (CI)	1.56 (1.39–1.76)		1.57 (1.40–1.76)	
p value	p<0.0001		p<0.0001	

CI, confidence interval; Gd^{+}, gadolinium-enhancing; IFN β-1a, interferon beta-1a; MRI, magnetic resonance imaging; NEDA, no evidence of disease activity; OCR, ocrelizumab.
